# Electrochemical Exfoliation of Graphite to Graphene-Based Nanomaterials

**DOI:** 10.3390/molecules27248643

**Published:** 2022-12-07

**Authors:** Michael Salverda, Antony Raj Thiruppathi, Farnood Pakravan, Peter C. Wood, Aicheng Chen

**Affiliations:** 1 Electrochemical Technology Centre, Department of Chemistry, University of Guelph, 50 Stone Road East, Guelph, ON N1G 2W1, Canada; 2 Zentek Ltd., 24 Corporate Court, Guelph, ON N1G 5G5, Canada

**Keywords:** electrochemical exfoliation, graphene oxide, expanded graphite, graphite, energy storage

## Abstract

Here, we report on a new automated electrochemical process for the production of graphene oxide (GO) from graphite though electrochemical exfoliation. The effects of the electrolyte and applied voltage were investigated and optimized. The morphology, structure and composition of the electrochemically exfoliated GO (EGO) were probed by scanning electron microscopy (SEM), transmission electron microscopy (TEM), X-ray diffraction (XRD), energy dispersive X-ray (EDX) spectroscopy, X-ray photoelectron spectroscopy (XPS), FTIR spectroscopy and Raman spectroscopy. Important metrics such as the oxygen content (25.3 at.%), defect density (I_D_/I_G_ = 0.85) and number of layers of the formed EGO were determined. The EGO was also compared with the GO prepared using the traditional chemical method, demonstrating the effectiveness of the automated electrochemical process. The electrochemical properties of the EGO, CGO and other carbon-based materials were further investigated and compared. The automated electrochemical exfoliation of natural graphite powder demonstrated in the present study does not require any binders; it is facile, cost-effective and easy to scale up for a large-scale production of graphene-based nanomaterials for various applications.

## 1. Introduction

Graphene nanomaterials have the potential to catalyze the green energy revolution as they have many important applications, including (but not limited to) energy conversion and energy storage [1,2,3,4,5,6,7,8,9,10,11,12,13,14]. To this end, reduced graphene oxide (rGO), heteroatom doped graphene, and three-dimensional (3D) graphene-based nanomaterial frameworks are being extensively investigated [15,16,17,18,19]. However, the implementation of their commercial applications has been largely limited to the lab scale as current synthesis methods are expensive and substantial quantities of chemical wastes are generated [20,21,22,23]. With increasing demands for energy storage materials that power portable electronics and electric vehicles, it is critical to consider the environmental implications associated with their production. The common routes to produce graphene-based nanomaterials, top-down and bottom-up strategies, are either too costly or detrimental to the environment (or both) to be economically viable for various applications that have been promoted [24,25,26]. Alternate approaches such as ECE may offer new opportunities as they require fewer chemicals and lower temperatures than traditional methods.

This work explores the feasibility of electrochemical exfoliation to produce graphene-based nanomaterials from graphite powder for industrial applications. Due to its high conductivity and 3D layered structure, graphite is an excellent candidate for ECE techniques to produce 2D graphene sheets [27,28,29,30,31,32,33,34]. The ECE of graphite has garnered considerable attention in recent years due to its potential as a scalable method for producing graphene-based nanomaterials from graphite. The form of the starting material is important to consider in ECE methods as they must have the capacity to withstand the ECE conditions. Graphite rods, graphite foils, and graphite powders have been investigated in the ECE process, with the most common form being graphite rods [35,36,37]. In general, solid graphite electrodes have been used as anodes in the electrochemical synthesis of graphene oxide, mainly relying on binding agents and various pre-treatments. This can be achieved by including polymeric binders, such as polyvinylidene fluoride, to form an electrode or the use of compressed porous bags that keep the graphite particles in contact with each other. However, introducing polymeric binders inevitably reduces conductivity and also likely introduces impurities [38,39]. Alternatively, graphite can be expanded with a chemical and thermal treatment to make expanded graphite (EPG), which may be compressed to form binder-free graphite foils [40].

**Scheme 1:**
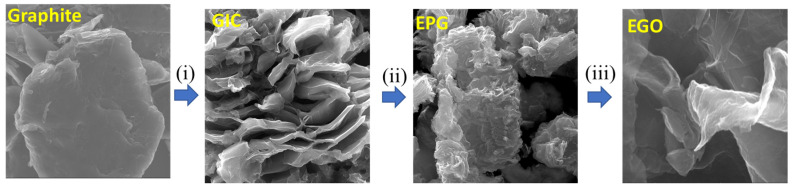
Overview of the conversion of natural graphite to electrochemically exfoliated graphene oxide: (**i**) chemical treatment of graphite to form GIC; (**ii**) thermal treatment of GIC to form EPG; (**iii**) electrochemical exfoliation of the EPG foil to produce EGO.

Scheme 1 illustrates the novel process we have developed in the present study, whereby graphite was chemically treated to form a graphite intercalation compound (GIC), which had a higher volume than the graphite. The formed GIC was then thermally expanded to produce EPG whose volume was dramatically increased compared to the GIC. Once expanded, the material can be compressed into foils and subjected to exfoliation conditions to produce electrochemically exfoliated graphene oxide (EGO).

## 2. Results and Discussion

**Figure 1:**
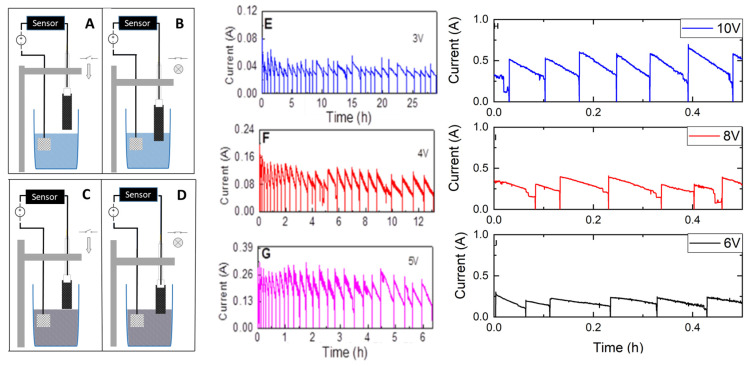
Principle of the automatic ECE system: (**A**) before ECE; (**B**) immersion of a fraction of the electrode; (**C**) the immersed portion was exfoliated; (**D**) re-immersion of a fraction of the electrode. Current and time plots recorded in 1.0 M NaOH during the ECE process at an applied voltage of: (**E**) 3.0 V, (**F**) 4.0 V, and (**G**) 5.0 V and in 0.5 M NaOH during the ECE process at an applied voltage of: (**J**) 6.0 V, (**I**) 8.0 V, and (**H**) 10.0 V.

Figure 1A–D illustrate the operation of the automatic ECE process. In Figure 1A, the system was at the open circuit, where the current was 0. The motor drove the electrode down to the solution with a pre-set height as shown in Figure 1B. Under the applied voltage, the submersed electrode was then consumed, and the current dropped to 0 as seen in Figure 1C. This process was repeated as shown in Figure 1D until a pre-set height had been traveled. Figure 1E–G display the current vs. time plots during the ECE of 1 cm × 5 cm EPG foils in 1.0 M NaOH under the applied voltages of 3.0, 4.0, and 5.0 V, respectively, showing a strong effect of the voltage. When the applied voltage was increased from 3.0 to 5.0 V, the generated current was increased and the ECE time was decreased from ~30 h to 6.5 h. The effect of the NaOH concentration on the ECE process was further investigated. Figure 1H–J display the exfoliation of a 1 cm × 5 cm EPG foil in 0.5 M NaOH, showing that a higher applied voltage was needed to make the process efficient with a lower NaOH concentration.

**Figure 2:**
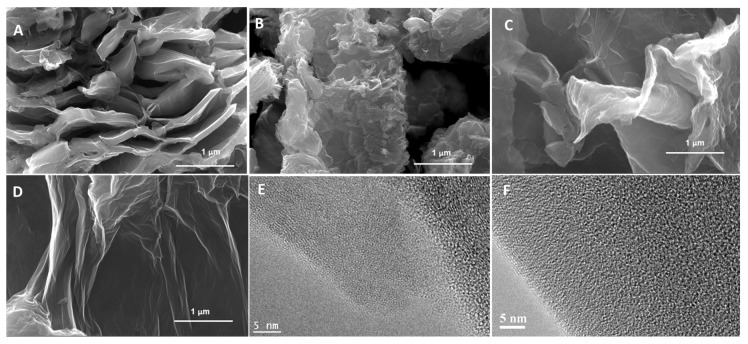
(**A**) Field emission scanning electron microscopy images of GIC before expansion, (**B**) EPG, (**C**) EGO, and (**D**) CGO. Transmission electron microscopic images of (**E**) EGO and (**F**) CGO.

Figure 2A illustrates the flaky structure of the formed GIC, showing an increased interlayer-distance compared to graphite. Following the thermal expansion, a worm-like structure was observed for the formed EPG (Figure 2B). Figure 2C displays the SEM image of the EGO which appears as a collection of 2 dimensional thin crumpled sheets, which is similar to the CGO (Figure 2D), indicating that the ECE process could effectively exfoliate the EPG foil to the GO-based material. The TEM images of the EGO and CGO are presented in Figure 2E,F, respectively, further demonstrating the similarities of the morphology between the EGO and CGO.

**Figure 3:**
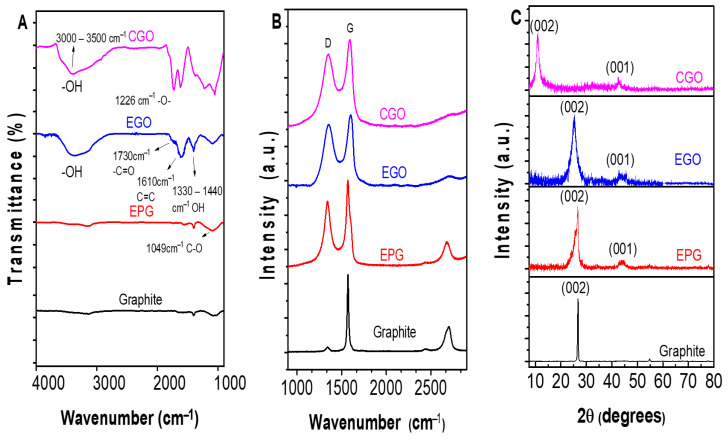
(**A**) Fourier Transform Infrared (FT-IR) spectra of graphite, (**B**) Raman spectra and (**C**) X-ray Diffraction (XRD) spectra of graphite, EPG, EGO, and CGO.

Fourier transform infrared (FT-IR) spectroscopy, Raman, X-ray diffraction, and X-ray photoelectron spectroscopy were employed to characterize the materials fabricated at different stages of the process. Figure 3A presents the FT-IR spectra of graphite, EPG, EGO, and CGO. The presence of the large broad peak at 3300 cm^−1^ was characteristic of -OH stretching, while the peak at ~1700 cm^−1^ was characteristic of carbonyl stretching [41]. The emergence of hydroxyl and carbonyl stretching suggested that many of these functionalities were generated during the ECE. For comparison, the IR spectrum of CGO was also included, showing that similar functional groups were introduced during the ECE process and in the course of the chemical exfoliation process.

The Raman spectra of graphite, EPG, EGO, and CGO are shown in Figure 3B, revealing the three characteristic bands (D, G, and 2D) at ~1360, ~1560, and ~2700 cm^−1^. The appearance of the D band was derived from the disorder-triggered scattering that arose from imperfections in the hexagonal structure of graphite. The G band originated from the in-plane stretching of the C=C bond vibrations of *sp*^2^-carbon or E_2_g vibration mode of graphite, whereas the D band was activated by the A_1_G breathing mode, and the 2D band emanated from the second order E_2_g vibration modes [42,43]. The relative intensities of the D and G bands can provide useful information regarding the surface defects present in the graphene-based material; a higher I_D_/I_G_ ratio is indicative of a higher defect density. Comparison of the I_D_/I_G_ ratios of the produced EGO to the graphite and EPG exhibited some key differences. The reduction of the 2D band intensity could be attributed to the introduction of defects, which suppressed the lattice vibration mode associated with the 2D peak. In addition, the decreased intensity of the 2D band indicated the exfoliation of graphite, whereas the reduced 2D band in the EPG indicated enlargement and a graphitic nature. Further, the EGO showed a broadened small hump-like 2D band, revealing the functionalization of graphite that is characteristic of GO [42,43]. The Raman spectrum of the EGO was well aligned with the spectrum of the CGO, further confirming that the ECE process can effectively exfoliate the EPG foil.

The XRD patterns of graphite, EPG, EGO, and CGO are displayed in Figure 3C. Graphite exhibited two distinct peaks corresponding to a hexagonal 002 plane and 100 basal plane at 26.70° and 44.46°, respectively. In comparison, the EPG showed a broad diffraction peak at 2Ɵ = 26.06°, which was due to an increased interlayer spacing as the result of the thermal expansion. Typically, the chemical oxidation of graphite to CGO resulted in a shift of the 002 peak in the XRD pattern to a lower 2θ (10.95°). This was due to the introduction of the oxygen functional groups into the graphite sheets, which increased the interlayer spacing. The EGO exhibited a weak and broader 002 peak at 25.0°. After the ECE process, the 002 peak was weaker and broader due to the anodic oxidation [44,45]. Since the XRD pattern of the EGO was featureless or weak (akin to graphene), the expected (002) peak ~10° was not noticeable. From the peak positions, the interlayer distances (d spacing) were estimated to be 0.337, 0.341, 0.354, and 0.807 nm for graphite, EPG, EGO, and CGO, respectively. Further, the crystallite sizes in the c-axis (Lc) were projected from the FWHM to be 24.0, 3.48, 2.79, and 4.48 nm for graphite, EPG, EGO, and CGO, respectively. The number of layers present was determined using Lc and d spacing to be 72, 11, 9, and 7 for graphite, EPG, EGO, and CGO, respectively. The EGO and CGO possessed a similar number of layers, showing that the thicknesses of the sheets were similar.

**Figure 4:**
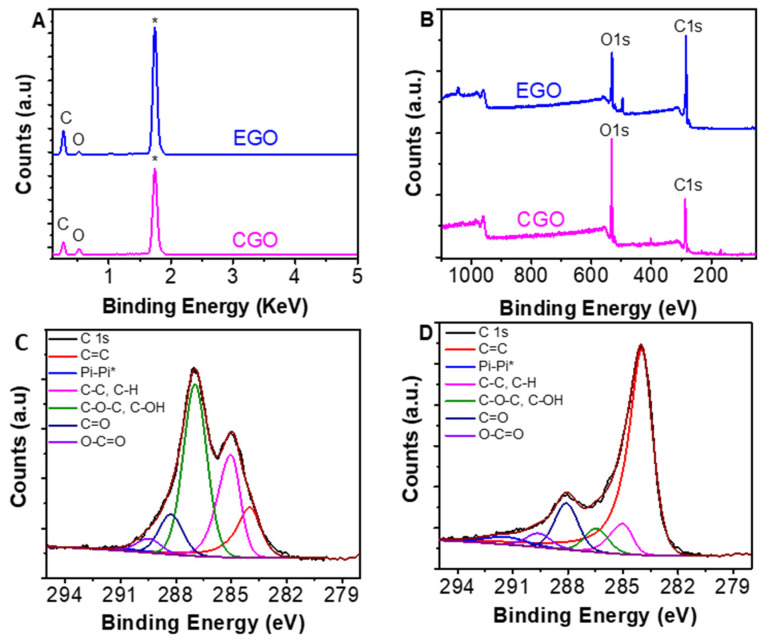
(**A**) Energy dispersive X-ray spectroscopy of EGO and CGO and (**B**) survey X-ray photoelectron spectroscopy of EGO and CGO and (**C**) high-resolution X-ray photoelectron spectroscopy (XPS) C1s for EGO and (**D**) high-resolution C1s for CGO.

To further elucidate the elemental composition and bonding environment, XPS characterization was performed on the EGO and CGO. For comparison, EDX spectra are presented in Figure 4A, where the strong carbon and oxygen peaks were observed, the (*) peak is attributable to the silicon background peak. The XPS survey spectra are presented in Figure 4B, while the deconvolutions of the high-resolution C1s XPS spectra of the EGO and CGO are displayed in Figure 4C,D, respectively. Notably, the peak at 288 eV (C=O) was the most intense oxygen-containing functional group, whereas for the CGO, the 287 eV (O-C-O and C-OH) peaks were the most intense. The relative intensities of the peaks corresponding to *sp*^2^ and *sp*^3^ carbon of the EGO suggested that the electrochemically derived material largely retained its *sp*^2^ nature compared to the CGO [46]. The atomic composition of the samples is listed in Table 1, showing that the oxygen content was increased of the EGO was much higher than that of the EPG, but was lower than that of the CGO.

**Figure 5:**
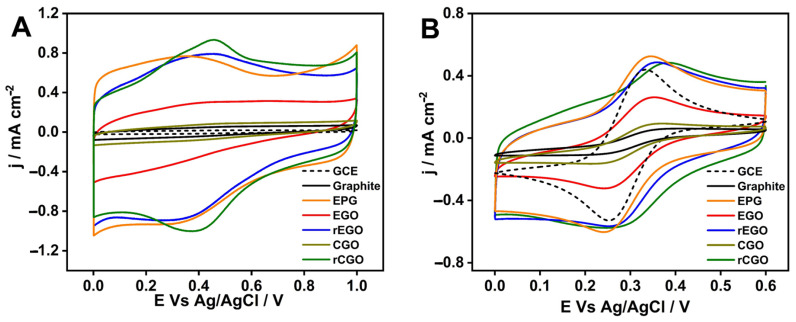
(**A**) Cyclic Voltammogram (CV) performed in 0.5 M H_2_SO_4_ at a scan rate 100 mVs^−1^; (**B**) CV performed at 50 mV s^−1^ in 0.1 M KCl + 5 mM potassium ferricyanide.

The electrochemically active surface area (EASA) and heterogenous electron transfer (HET) capability were studied to understand the differences in the electrochemical properties of graphite, EPG, EGO, CGO, reduced CGO (rCGO), and reduced EGO (rEGO). The rEGO and rCGO were obtained by electrochemical (EC) reduction of EGO and CGO at −1.6 V vs. Ag/AgCl in a 7.4 pH phosphate-buffered solution (PBS) for 600 s, respectively. Figure 5A displays the cyclic voltammograms recorded in a 0.5 M H_2_SO_4_ solution at the scan rate of 100 mVs^−1^. The area of the CVs is related to the electrochemical capacitance. For comparison, the CV of the smooth GCE substrate was also included as the dotted line. The CV of the graphite was slightly larger than that of the GCE due to its low surface area. The EPG exhibited a significantly greater capacitance compared to graphite and EGO, which could be attributed to the increase of the EASA and its high conductivity. Further, the EGO and CGO exhibited a small capacitance, which could be attributed to their poor conductivity due to the introduction of oxygen-containing functional groups. However, the EGO possessed a much higher capacitance than the CGO, which could be explained through its increased conductivity due to the smaller proportion of oxygen-containing functional groups [47], which is consistent with the EDX results shown in Table 1. Figure 5B shows the redox probe cyclic voltammetric (CV) curves of the glassy carbon electrode (GCE), graphite, EPG, EGO, rEGO, CGO, and rCGO recorded in 0.1 M KCl containing 5 mM K_3_[Fe(CN)_6_], where the potential was measured with respect to the Ag/AgCl reference electrode. The difference between the oxidation and reduction peak potentials (ΔEp) is an indicator, in which, as the difference increases, the rate of the electron transfer is slower and vice versa. As seen in Table 2, the ΔEp of the GCE, graphite, EPG, EGO, rEGO, CGO, and rCGO was found to be 78, 149, 96, 103, 84, 171, and 104 mV, respectively. The GCE exhibited a faster HET performance with the smallest ΔEp, whereas the EGO exhibited a lower ΔEp compared to the CGO, which supported the higher conductivity of the EGO. The reduced conductivity of the EGO and the CGO was also likely responsible for the blocking of the ferricyanide response, as electron transfer rate [48]. After the EC reduction, the formed rEGO and rCGO exhibited a significantly higher capacitance than the EGO and CGO, which could be attributed to the reduction of functional groups and increased conductivity. The accessible surface area of the EGO, rEGO, CGO, and rEGO could be approximated from the EASA, which might be calculated from the double-layer capacitance (C_dl_) measurements via cyclic voltammetry at different scan rates varied from 5 to 100 mVs^−1^.

**Figure 6:**
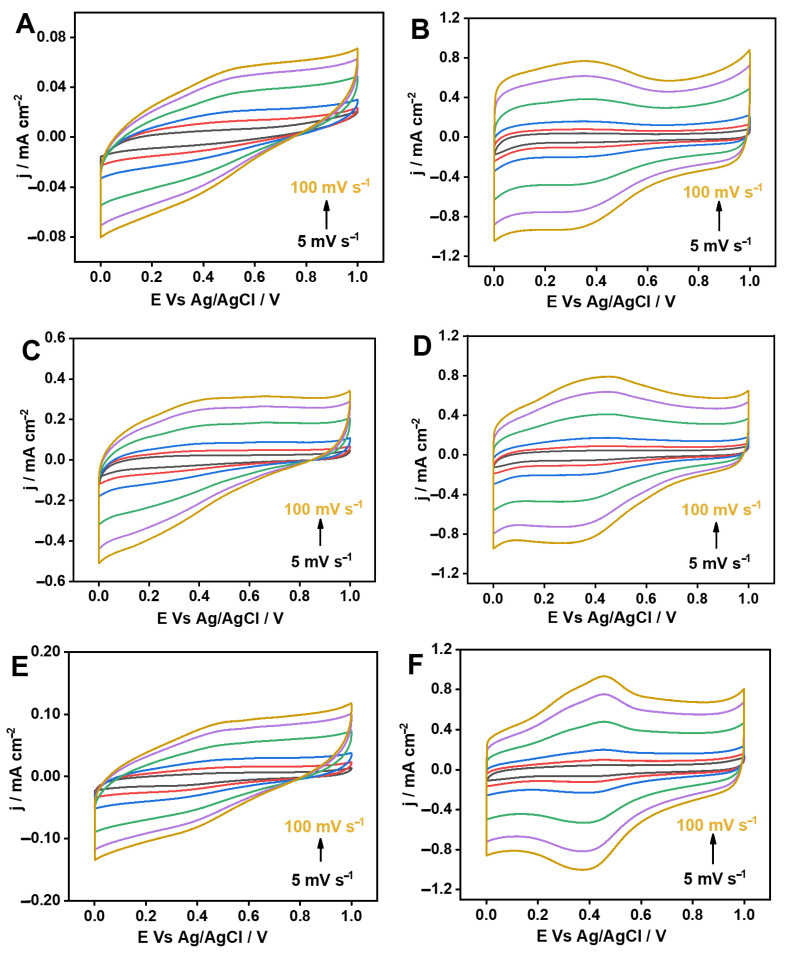
Scan rate effect studies in 0.5 M H_2_SO_4_ of (**A**) graphite, (**B**) EPG, (**C**) EGO, (**D**) Electrochem-ical reduced EGO (rEGO), (**E**) CGO, and (**F**) Electrochemical reduced CGO (rCGO).

The C_dl_ was assessed from the linear regression slope between the current density differences [Δj/2 = (ja − jc)/2] in the middle of the potential window of the CV curves recorded at the different scan rates. The corresponding cyclic voltammograms are presented in Figure 6A–F and were recorded in H_2_SO_4_ by varying the scan rate from 5 to 100 mVs^−1^. Similar behaviours were observed for graphite (Figure 6A), EGO (Figure 6C) and CGO (Figure 6E), which can be attributed to the low surface area of the graphite and the low conductivity of the EGO and CGO due to the high oxygen content. In contrast, redox peaks were seen for EPG (Figure 6B), rEGO (Figure 6D) and rCGO (Figure 6F) at the high scan rate, which is consistent with the results shown in Figure 5A.

**Figure 7:**
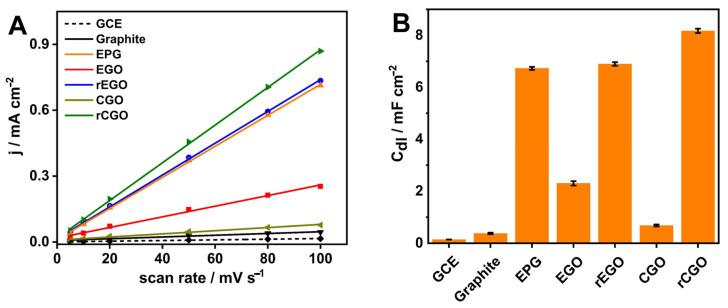
(**A**) Double layer capacitance determination from scan rate studies, (**B**) A bar chart of dou-ble layer capacitance values of all samples.

The estimated C_dl_ was normalized to the geometric area of the smooth GCE substrate (0.07 cm^2^). As summarized in Table 2, the C_dl_ values of the EGO, rEGO, CGO, and rCGO were determined to be 2300, 6900, 680.0, and 8170 µF cm^−2^, respectively, shown in Figure 7A,B. The C_dl_ results again confirmed that EGO had a higher capacitance than CGO. However, after the EC reduction, the formed rEGO and rCGO exhibited similar electrochemical behaviors. The EPG exhibited faster HET, similar to rEGO and rCGO. In addition, an increased double layer current could be observed for the EPG, rEGO, and rCGO compared to the others, which could be attributed to their high conductivity and larger active surface area [48]. HET studies also confirmed a similar behaviour for the EGO and CGO. EASA and HET kinetics are fundamental electrochemical attributes used to evaluate nanomaterials for various electrochemical sensor, catalyst, and energy storage applications. The EGO and CGO exhibited similar capacitance and HET behaviours [49].

## 3. Materials and Methods

### 3.1. Materials

High-purity graphite powder (Albany graphite deposit) was provided by Zentek Ltd. (Guelph, ON, Canada). Sulfuric acid (98%), iron (III) chloride hexahydrate (99.9%), sodium hydroxide (99%) hydrogen peroxide (30%), analytical grade reagents (phosphoric acid (85%), and potassium permanganate (≥99.0%) were used as received without further purification. Pure water (18.2 MΩ cm, via a Nanopure^®^ Diamond™ UV water purification system) was used in the preparation of the aqueous solution.

### 3.2. Synthesis of the Expanded Graphite

In an exemplar synthesis of expanded graphite (EPG), graphite (1.00 g) was combined with sulfuric acid (15.0 mL, 98% *w*/*w*) and phosphoric acid (4.0 mL, 85% *w*/*w*), and the resultant reaction mixture was magnetically stirred at 0 °C with an ice bath. Potassium permanganate (2.00 g) was then added to the reaction mixture, which was subsequently stirred for 30 min, after which iron (III) chloride hexahydrate (0.50 g), which was found to enhance the expansion, was added to the reaction mixture and stirred for an additional 1 h. The reaction mixture was then separated by centrifugation, with the sedimented material being collected and dried in an oven at 50 °C for 24 h. The expansion of the GIC was found to be optimized at the temperature of 640 °C. The dark grey, dry, and crumbly intercalated graphite compound was then thermally treated at the aforementioned temperature for 3 min to form the EPG.

### 3.3. Synthesis of Electrochemically Exfoliated Graphene Oxide

In a typical synthesis of EGO, the EPG powder was compressed into a 5.0 cm × 1.0 cm thin foil through a hydraulic press. A two-electrode system was employed for the electrochemical exfoliation, where the EPG foil served as the working electrode and a platinum mesh was used as the counter electrode. Sodium hydroxide solutions with different concentrations were used as the electrolyte. An automation device was developed for the control of the ECE process. Following the completion of the exfoliation, the solution was neutralized using HCl to pH ~2 to separate the formed EGO from the NaOH electrolyte. After centrifuge, the obtained EGO was rinsed with ethanol and dried in an oven at 50 °C overnight.

### 3.4. Automation of the ECE Process

To facilitate a reproducible and scalable exfoliation process, a current-based anode feeder was designed and implemented as an attachment module for a typical potentiometry workstation. When connected in series with the exfoliation circuit, the feeder monitored the total current of the system and adjusted the exposed area of the anode. The use of this module in tandem with the exfoliation process allowed for the control of the electrolyte-exposed surface area of the electrode, which was limited to manual adjustments at specific time intervals. As such, by enabling the control of this parameter in situ, a major experimental and scalability barrier was eliminated.

The system was built upon the Arduino architecture, with a dedicated hull-effect sensor that provided a signal to the main microcontroller, which directed the reaction parameters. A custom elevator was designed and fabricated using a leadscrew and stepper motor to translate the rotational motion of the motor to the linear actuation of the anode platform. In a typical experiment, the anode was aligned with the cell and the experiment was software initiated. The elevator continuously lowered the electrode into the electrolyte until the circuit was established and a specified minimum current was measured. The exfoliation proceeded as soon as a connection between the electrode and the electrolyte was established. When the area of the electrode consumed as the graphite layers were liberated, the current decreased proportionally to the shrinking electrode area, which subsequently generated a signal to lower the elevator and immerse a pristine portion of the electrode into the cell.

### 3.5. Synthesis of Graphene Oxide

For comparison, a typical chemically synthesized graphene oxide (CGO) was prepared using a modified Hummers’ method. Pure graphite (2.00 g) was added into 200 mL of 9:1 sulfuric acid/phosphoric acid (*v*/*v*) and stirred for 2 h, after which potassium permanganate (9.00 g) was added. The mixture was then further stirred for 15 h and subsequently placed in an ice bath, followed by the addition of 30% H_2_O_2_ (2.5 mL). The CGO was then rinsed with hydrochloric acid and ethanol and collected via centrifugation [3].

### 3.6. Surface Characterization

The morphology of the synthesized materials and their elemental compositions were probed by the field emission scanning electron microscopy (FE-SEM) (FEI Quanta FEG 250 SEM), the high-resolution transmission electron microscopy (HR-TEM) (FEI Tecnai F30 electron microscope, using a 200 kV accelerating voltage) and the energy dispersive X-ray (EDX) spectroscopy (FEI Inspect S50 SEM with an attached EDX detector with ±0.1 at.%). The Raman spectra were recorded using a Renishaw Raman spectrometer at 50× magnification with a λ = 532 nm laser source. The X-ray diffraction patterns were obtained via a Panalytical PW1050-3710 diffractometer with Cu Kα (λ = 1.5405 Å) as an X-ray source. The FTIR spectra were measured with a Thermo Scientific FTIR spectrometer. X-ray photoelectron spectroscopic (XPS) measurements were performed using a Scienta Omicron system with an Al Kα X-ray source and 700 µm spot size.

### 3.7. Electrochemical Characterization

The electrochemical studies were performed using cyclic voltammetry with a CHI 660E potentiostat and a three-electrode cell. A glassy carbon electrode (GCE) (surface area = 0.07 cm^2^) was used as the substrate to coat the synthesized materials and served as the working electrode, while an Ag/AgCl in saturated KCl and a platinum coil were used as the reference electrode and the counter electrode, respectively. To make the coatings 4.0 mg of the synthesized materials was dispersed into a mixture of 600 µL of pure water, 300 µL of isopropanol and 100 µL of Nafion (5 wt.%), and 3.0 µL of the prepared ink was cast onto a clean GCE. The prepared GCEs were then allowed to dry overnight before being used for the electrochemical studies.

## 4. Conclusions

In summary, we have demonstrated a new approach for the efficient conversion of graphite to graphene-based nanomaterials by employing the electrochemical exfoliation. Through a thermally expanded graphite intermediate, the EPG foil was made and subsequently electrochemically exfoliated to produce EGO comparable to chemically produced graphene oxide. Various surface characterization techniques and spectroscopic methods were employed to study the layer structure, composition and functional groups of the formed EGO, showing the high quality of the GO produced by the ECE process. After the electrochemical reduction, the formed rEGO and rCGO exhibited high conductivity and large capacitance. This proposed ECE process was further tested with 5 × 5 cm^2^ foils; similar surface characterization results and electrochemical properties of the EGO produced from the 5 and 25 cm^2^ foils were obtained, demonstrating the high scale-up capability. The automated ECE process described in this study is promising for the large-scale production of graphene-based nanomaterials from graphite for various environmental, energy and medical applications.

## Figures and Tables

**Table 1:** Energy-dispersive X-ray spectroscopic data of the materials.

Material	Carbon (at.%)	Oxygen (at.%)
Graphite	100.0	0.0
EPG	91.9	7.4
EGO	74.7	25.3
CGO	65.6	35.4

**Table 2:** Comparison of electrochemical results of the GCE substrate, graphite, EPG, EGO, rEGO, CGO and rCGO.

Electrode	Ea mV	Ec mV	ΔE_p_ mV	C_dl_ µF cm^−2^	R^2^
GCE	331.6	254.0	77.6	144.0	0.9942
Graphite	361.6	212.5	149.1	379.6	0.9721
EPG	343.1	246.7	96.4	6730	0.9996
EGO	349.3	246.7	102.6	2300	0.9936
rEGO	366.9	282.6	84.4	6900	0.9998
CGO	380.1	209.6	170.5	311.2	0.9979
rCGO	377.2	273.6	103.6	8170	0.9995

## Data Availability

Data is contained within the article.

## Sample Availability

Samples of the compounds are available from the authors.
